# What do physicians know about homosexuality? Translation and adaptation of Knowledge about Homosexuality Questionnaire

**DOI:** 10.1590/S1679-45082018AO4252

**Published:** 2018-09-11

**Authors:** Renata Corrêa-Ribeiro, Fabio Iglesias, Einstein Francisco Camargos

**Affiliations:** 1 Programa de Pós-Graduação em Ciências Médicas, Faculdade de Medicina, Universidade de Brasília, Brasília, DF Brazil; 2 Programa de Pós-Graduação em Psicologia Social do Trabalho e das Organizações, Universidade de Brasília, Brasília, DF, Brazil

**Keywords:** Homosexuality, Health knowledge, attitudes, Physicians, Surveys and questionnaires, Homossexualidade, Conhecimentos, atitudes e prática em saúde, Médicos, Inquéritos e questionários

## Abstract

**Objective:**

To adapt the Knowledge about Homosexuality Questionnaire to Brazilian Portuguese, and to assess knowledge of heterosexual physicians on homosexuality.

**Methods:**

The following steps for cultural adaptation were made: translation by two independent evaluators, translation synthesis, and evaluation of semantic properties by the target population, followed by the development of a pilot study and administration of the instrument to 224 heterosexual physicians working in the Brazilian Federal District.

**Results:**

The mean number of correct answers in the questionnaire was 11.8 (SD=2.81) out of 18 items, *i.e.,* 65.5%. Catholic and evangelical physicians gave a significant lower number of correct answers compared with those who believed in other religions or who did not believe in any religion (p=0.009), and 40% of sample did not know that homosexuality is not considered a disease.

**Conclusion:**

This study adapted the American instrument entitled Knowledge about Homosexuality Questionnaire and provided evidence for its validation in Brazil, revealing physicians' lack of knowledge about several aspects related to homosexuality. The findings of this study may help in guiding improvements in medical training and practice.

## RESUMO

**Objetivo:**

Realizar a adaptação para o Brasil do inventário *Knowledge about Homosexuality Questionnaire* e avaliar o conhecimento de médicos heterossexuais sobre a homossexualidade.

**Métodos:**

Foram realizadas as etapas de adaptação cultural: tradução por dois avaliadores independentes, síntese da tradução e avaliação das propriedades semânticas pela populaçãoalvo, estudo piloto e aplicação do inventário em 224 médicos heterossexuais, atuantes no Distrito Federal.

**Resultados:**

A média de acerto do questionário foi de 11,8 (DP=2,81) dos 18 itens, ou seja, 65,5%. Médicos católicos e evangélicos acertaram significativamente menos itens quando comparados aos que adotavam outra ou nenhuma religião (p = 0,009), e 40% da amostra não sabia que a homossexualidade não é considerada doença.

**Conclusão:**

Esse estudo adaptou, com evidências de validade para o Brasil, o inventário estado-unidense *Knowledge about Homosexuality Questionnaire* e revelou desconhecimento de vários aspectos relacionados à homossexualidade, o que pode orientar melhor a formação e a prática médica.

## INTRODUCTION

Several studies have shown differences in the prevalence of diseases and risk factors in the lesbian, gay, bisexual and transgender (LGBT) [population in comparison to heterosexuals, such as: a higher rate of smoking;^(^ ^1^ ^–^ ^4^ ^)^ excessive alcohol use;^(^ ^1^ ^,^ ^2^ ^,^ ^4^ ^)^ obesity;^(^ ^1^ ^,^ ^2^ ^,^ ^4^ ^,^ ^5^ ^)^ drug use;^(^ ^3^ ^,^ ^6^ ^–^ ^8^ ^)^ and cardiovascular diseases.^(^ ^1^ ^)^ One possible explanation for that, according to the Minority Stress Model, is that minorities are more susceptible to health problems because they experience chronic stress due to social stigmatization.

The discrimination sexual minorities suffer when seeking medical attention has been brought to light by a few medical societies, such as the American Geriatrics Society. In 2015 this association reported the following evidence regarding healthcare for the LGBT public: refusal of certain medical treatments; incorrect assumption that the patient is heterosexual; refusal to accept certain companions chosen by the patient during hospitalization offensive and derogatory statements. These factors can lead the LGBT population to delay or avoid seeking medical attention for fear of being subjected to discrimination.^(^ ^9^ ^,^ ^10^ ^)^

A recently published systematic review assessed the relation of the LGBT public with the healthcare system and showed several issues, such as lack of training for healthcare professionals, who have difficulty approaching sexuality related matters and operate under a heteronormativity premise; barriers and institutionalized prejudiced practices; demands of sexual minorities that are not met, which increases the risk of mental illness, suicide, cancer and a higher susceptibility to sexually transmitted diseases; increased internalized homophobia due to a perceived rejection by medical professionals who should treat and welcome all patients; fear of accessing medical services, which leads to avoidance or delays in treatment; concealment of sexual orientation; increase of self-medication or seeking information about treatment at drugstores, in magazines, with friends or online; late seeking of medical services in extreme or emergency cases; experiencing of homophobic statements, humiliation, ridicules and breach of confidentiality.^(^ ^11^ ^)^

Moreover, studies have shown the relation between lack of knowledge about homosexuality and negative or prejudiced attitudes from physicians, undergraduate medical students and other healthcare professionals regarding the LGBT population.^(^ ^12^ ^–^ ^16^ ^)^

The lack of studies in Brazil that evaluate physicians’ knowledge about homo- and bisexuality may be partly explained by the lack of specific questionnaires that evaluate the knowledge of elementary aspects of homosexuality. We believe that having questionnaires available in Portuguese could make it possible to evaluate physicians’ knowledge and implement policies regarding medical services for the LGBT population. The Knowledge about Homosexuality Questionnaire (KHQ), one of the most frequently used questionnaires in the world, is an instrument about general knowledge regarding homosexuality.^(^ ^17^ ^)^

## OBJECTIVE

To adapt the Knowledge about Homosexuality Questionnaire to Brazil and evaluate the knowledge of heterosexual physicians about homosexuality.

## METHODS

### Participants

The recruiting method chosen was the “snowball technique”, using a promotion of the research and inviting physicians via e-mail, social networks, and societies of different specialties. We included active physicians working in the Federal District of Brazil who are self-declared heterosexuals. The sample calculation followed the recommendations of having ten participants for each observable variable.^(^ ^18^ ^,^ ^19^ ^)^ Therefore, considering the original inventory presents 20 items, we sought a sample of at least 200 valid cases. The exclusion criteria were not disclosed to the participants in order to avoid inhibitions or embarrassing situations, especially with regards to information about sexual orientation. In these cases, the physicians were only excluded from the sample in the data analysis process.

Initially, 306 physicians of both genders responded to the survey. Of those, 45 were excluded for not completing their participation, 17 for not working in the Federal District, 13 for not being heterosexual, and 7 for being retired.

### The instrument

The KHQ addresses central and objective aspects of homosexuality, distinguishing it from other characteristics it is often erroneously associated to. This instrument evaluates knowledge and not beliefs or opinions, focusing on the cognitive and not emotional or behavioral elements of the answers.^(^ ^17^ ^)^

For this study we decided to use the version of KHQ modified by Koch, in which two items were removed (due to the results of a validation process in their sample) and the option “I do not know” was introduced to allow a more reliable assessment of knowledge. This modified version has 18 true/false items regarding knowledge about homosexuality and generates a score of zero to 18, where 18 means 100% correct.^(^ ^20^ ^)^ Thus, the higher the number of correct answers, the higher the knowledge. The number of correct items was converted into a percentage of correct answers: no correct answers = 0% and 18 correct answers = 100%.

A study carried out the United States, in 2003, used this version modified by Koch (18 items) with 408 professors and had an average correctness of 9.71 (standard deviation – SD of 3.49), that is, 54% of items with Cronbach's alpha of 0.72.^(^ ^21^ ^)^ The construct validity evidence showed that the respondents with a higher level of education obtained the highest scores and a sexual education professor was accurate in all items.^(^ ^22^ ^)^

### Procedures

This study followed all procedures to ensure accuracy of contents of the original instrument, which included translation of the original language into the target language by two evaluators; synthesis of the translated versions; analysis of the final version by two expert judges; semantic evaluation by the target audience; and a pilot study.^(^ ^19^ ^)^

The translation was done by two people separately: an experienced researcher in the area of instrument adaptation, and a professional with no ties to academia, both of whom are fluent in English. The synthesis of the two translations was conducted by a group of three university lecturers of psychometrics, with a vast knowledge on instrument adaptation, who met to evaluate each item of the two translated versions and then conflate them into one new version. The discussion over the final version included the authors and one of the psychometrics researchers.

Lastly, the final version was presented to the target audience for the penultimate stage of the adaptation process. In the stage, the target audience of 22 physicians was invited to evaluate the questionnaire's items to determine if the items were clear or not, and to suggest alterations for items they believed were not clear. The physicians were chosen for convenience – several physicians were sent invitations and those who agreed to participate in this stage were selected. At the end of the stage of instrument application to this group of physicians who formed the target audience, their suggestions were evaluated by the researchers and the psychometrist, so that a final decision could be reached regarding which suggestions would be taken, thus arriving at the final version of both instruments. This final version was then applied to the group of physicians from the pilot study.

After the alterations were made, the instrument was made available on an online data collection platform, so we could conduct the quantitative pilot study, which included 42 physicians. We also included items about the participants’ demographic data such as age (in years), sex (male/female), religion (catholic, evangelical, spiritist, none, or other), marital status (single, married/common-law marriage, separated/divorced, widow/er), professional practice (acting physician, resident, retired, non-acting), and sexual orientation (heterosexual, homosexual, bisexual, none of the above, I do not know, other). The only change between the pilot study and the next stage of the study data collection was that a question was added about the place of work (in the Federal District, outside the Federal District, in and outside the Federal District). The reason for that added questions was the risk that, because we used the “snowball technique”, the survey would reach physicians who do not work in the Federal District, which would hinder the sample's characterization and consistency.

Data collection took place between March and September 2016. The study was authorized by the Research Ethics Committee of the organization under protocol n° 1.339.076, CAAE: 49275115.0.0000.5558, and the use of an informed consent was waived, since it could deter or identify the participants. The request for a limited set of sociodemographic data helped ensure the study's anonymity, even to the authors.

### Data analysis

In the evaluation process, all items were transformed so that all incorrect or “I do not know” answers received a score of 0, and correct answers received a score of 1. Thus, the higher the average obtained, the higher the number of correct answers.

The data were analyzed with descriptive statistics to obtain frequency distributions through the software Statistical Package for the Social Sciences (SPSS), version 21. After that, we performed inferential analyses through Spearman's correlation test to check if there was a relation between KHQ's total score and other variables studied, and we used Student's t test for independent samples.

## RESULTS

In the stage of evaluation by the target audience (fourth stage of the adaptation process), we evaluated 22 physicians who responded to the instrument and were asked about clarity of items. Some suggestions in the evaluation by the target audience were not contemplated in the instrument's final version when the suggested “lack of clarity” was not due to a semantic or idiomatic problem but to the respondent's lack of knowledge on elementary aspects of homosexuality.

Item 1, which stated “a child who engages in homosexual behavior will become a homosexual adult” was classified as unclear by three out of the 22 participants. This item refers to some sexual games children play with other children of the same gender, but some members of the target audience are not aware of this type of activity among children. Therefore, it was suggested that the item be changed to “displays homosexual behavior”. However, this alteration would change the meaning of the item and could raise doubts about the aspects related to gender manifestation (*e.g*. boys with feminine behaviors). For that reason, this suggestion was not accepted. Even though we kept the semantic characteristics of the item, and questions were raised regarding the use of the term “homosexual” referring to children, for whom most practices are more guided by curiosity and a wish to belong to the group than by affective/sexual desire. Even if we had considered that the term “sexual games among children of the same gender” would make the item even clearer, this change would mean much modification from the original version and so we decided against it, considering the term “behavior” belongs to the dimension of practices and attitudes and not necessarily desire.

Item 5 in the version before evaluation by the target audience stated that “sexual orientation is established at an early age”. Five physicians pointed out that “at an early age” is quite imprecise, and the suggested alteration was accepted. The item was then rewritten to “sexual orientation is established at an early age (during adolescence or even before that)”, which is in accordance with the American Academy of Pediatrics.^(^ ^23^ ^)^

Item 12 resulted in significant changes from the original which read: “Kinsey and many other researchers consider sexual behavior as a continuum from exclusively homosexual to exclusively heterosexual.” It was altered to “Many researchers consider sexual behavior as a continuum that goes from exclusively homosexual to exclusively heterosexual.” This alteration was done because researcher Alfred Kinsey, despite his great importance in the field of human sexuality studies in the 1940s and 1950s, is not widely known in Brazil, except by professionals who have studied about human sexuality. Therefore, the item was altered, but was still classified as unclear by half of the target audience (11 physicians) regarding the term “continuum”. We decided to try to provide a more comprehensive idea of gradation, as suggested by the target audience, without deconfiguring the original item, which required a certain level of knowledge about sexuality and could therefore be unclear to some professionals. The final version of Item 12 was: “Many researchers consider sexual behavior to be a continuum that can vary from exclusively homosexual to exclusively heterosexual.” By adding the phrase “that can vary from”, the word continuum was understood as an idea of gradation between two opposites.

Item 13, “a homosexual person's gender identity does not agree with their biological sex”, was not understood by seven physicians. They suggested we added an explanation of “gender identity”, which is a clear example that the item's lack of clarity could be due to a lack of knowledge on the subject by part of the target audience and not due to flaws in the adaptation process.

Item 18, which originally read “Recent research has shown that homosexuality may be linked to chromosomal differences” was adapted to “Recent research has shown that homosexuality may be related to genetic differences” as suggested by the researcher, in light of the current knowledge that suggested a relation between homosexuality and genetic alterations linked to the X chromosome.^(^ ^24^ ^)^ In the evaluation by the target audience, no alterations were suggested for this item.

After the adaptation phase, the instrument's final version was added to the online platform for the second stage of the research (sample evaluation).

The final sample included 224 heterosexual physicians who worked in the Federal District, aged between 24 and 72 years (mean age 42.2 years; SD=9.5 years), 149 (66.5%) of whom were female (Table 1).

**Table 1:** Characteristics of the study participants

Variables		n (%)	Pearson's correlation coefficient
Age groups, years			
	<34	49 (21.9)	0.0072
	35-44	91 (40.6)	
	45-54	61 (27.2)	
	55-64	20 (8.9)	
	65-74	3 (1.3)	
Sex			
	Female	149 (66.5)	-0.0137
Position			
	Physician (staff)	208 (92.9)	
	Physician (resident)	16 (7.1)	
Marital status			-0.0448
	Single	31 (13.8)	
	Married/common law marriage	174 (77.7)	
	Separated/divorced	18 (8.0)	
	Widow(er)	1 (0.4)	
Religion			0.1504*
	Catholic	102 (45.5)	
	Spiritist	41 (18.3)	
	Evangelic	27 (12.1)	
	None	44 (19.6)	
	Others	10 (4.5)	

* p<0.05.

Of 261 physicians who completed the research, 248 (95%) declared themselves heterosexual.

The mean of correct answers was 11.8 (SD 2.81) of 18 items, that is, 65.5% of all items. The number of correct answers ranged between 2 and 18 (alpha=0.61). The ‘catholic’ and ‘evangelical’ categories combined had a significantly lower number of correct items, with a mean of 11.43 (SD 2.77) in comparison to ‘other’ or ‘no religion’, whose correct items had a mean of 12.42 (SD 2.78; *t*(222)=2.63; p=0.009, d=0.47 - variables not adjusted for age). No differences were found in answers between male and female physicians, *t*(222)<1, p=ns.

Item 14 had the highest percentage of wrong answers. It read: “Historically, almost every culture has evidenced widespread intolerance toward homosexuals, viewing them as sick or as sinners”. Only 16.5% of participants marked the statement as false, which is the correct answer. Item 17 had the highest number of correct answers. It stated that “Bisexuality may be characterized by sexual behaviors and/or responses to both sexes”, and was marked as true, which is the correct answer, by 96.6% of participants (Table 2). Item 2, which states “There is a good chance of changing homosexual people into heterosexuals”, revealed that 19.6% of physicians did not know if that is in fact possible, and 7.6% thought it was possible. Item 6, “Homosexuality is an illness, was responded to with ‘I do not know’ by 34.4% of participants and ‘yes’ by 4.9%.

**Table 2:** Response pattern to the Knowledge about Homosexuality Questionnaire

Items		n (%)
1. A child who engages in homosexual behavior will become a homosexual adult		
	True	9 (4.0)
	False*	180 (80.4)
	I do not know	35 (15.6)
2. There is a good chance of changing homosexual people into heterosexuals		
	True	17 (7.6)
	False*	163 (72.8)
	I do not know	44 (19.6)
3. Most homosexuals want to be members of the opposite sex		
	True	11 (4.9)
	False*	172 (76.8)
	I do not know	41 (18.3)
4. Some church denominations oppose legal and social discrimination against homosexual men and women		
	True*	136 (60.7)
	False	22 (9.8)
	I do not know	66 (29.5)
5. Sexual orientation is established at an early age (during adolescence or even before that)		
	True*	166 (74.1)
	False	23 (10.3)
	I do not know	35 (15.6)
6. According to the American Psychiatry Association, homosexuality is an illness		
	True	11 (4.9)
	False*	136 (60.7)
	I do not know	77 (34.4)
7. Homosexual males are more likely to seduce young men than heterosexual males are likely to seduce young girls		
	True	16 (7.1)
	False*	150 (67.0)
	I do not know	58 (25.9)
8. Gay men are more likely to be victims of violent crime than the general public		
	True*	178 (79.5)
	False	23 (10.3)
	I do not know	23 (10.3)
9. A majority of homosexuals were seduced in adolescence by a person of the same sex, usually several years older		
	True	27 (12.1)
	False*	124 (55.4)
	I do not know	73 (32.6)
10. A person becomes a homosexual (develops a homosexual orientation) because he/she chooses to do so		
	True	72 (32.1)
	False*	121 (54.0)
	I do not know	31 (13.8)
11. Homosexuality does not occur among animals (other than human beings)		
	True	11 (4.9)
	False*	183 (81.7)
	I do not know	30 (13.4)
12. Many researchers consider sexual behavior to be a continuum that can vary from exclusively homosexual to exclusively heterosexual.		
	True*	160 (71.4)
	False	12 (5.4)
	I do not know	52 (23.2)
13. A homosexual person's gender identity does not agree with his/her biological sex		
	True	91 (40.6)
	False*	100 (44.6)
	I do not know	33 (14.7)
14. Historically, almost every culture has evidenced widespread intolerance toward homosexuals, viewing them as “sick” or “sinners”		
	True	154 (68.8)
	False*	37 (16.5)
	I do not know	33 (14.7)
15. Heterosexual men tend to express more hostile attitudes toward homosexuals than do heterosexual women		
	True*	165 (73.7)
	False	17 (7.6)
	I do not know	42 (18.8)
16. “Coming out” is a term that homosexuals use for publicly acknowledging their homosexuality		
	True*	198 (88.4)
	False	16 (7.1)
	I do not know	10 (4.5)
17. Bisexuality may be characterized by sexual behaviors and/or responses to both sexes		
	True*	217 (96.9)
	False	4 (1.8)
	I do not know	3 (1.3)
18. Recent research has shown that homosexuality may be related to genetic differences		
	True*	69 (30.8)
	False	35 (15.6)
	I do not know	120 (53.6)

* Correct answer.

Source: Koch CA. Attitudes, Knowledge, and anticipated behaviors of presence teachers toward individuals with different sexual orientations Washington DC: George Washington University; 2000.^(^^20^^)^

Item 10, “a person becomes a homosexual because he/she chooses to do so”, was marked as ‘true’ by 32.1% of physicians, and 13.8% of them said they did not know. That showed that half the physicians were unaware of the several biopsychosocial aspects related to homosexuality and believe it to be an individual choice.

## DISCUSSION

Differences regarding health status among heterosexuals and non-heterosexuals are well described in the literature from the United States, motivating policies of specific care, committees for training and refresher courses for physicians of different specialties. In Brazil, however, there are no studies that use specific inventories (instead of general sexuality aspects) to evaluate the knowledge of physicians on homosexuality. That leaves the literature with a significant gap of information that could be translated into healthcare policies and professional training.

Even during the stage of instrument adaptation, the process of evaluating the target audience was complex because this tool is an inventory of knowledge and not of opinions, beliefs or behavior reports. The target audience often showed a lack of clarity regarding some items, which, in reality, showed the physicians’ lack of knowledge on the subject, but no flaws in the adaptation process.

The two items which had the highest number of correct answers in our study were also the ones with the most correct answers in a study conducted with high school teachers in the United States, which demonstrates that this knowledge is not restricted to the field of health. The two items in question are about the concept of bisexuality and the term “coming out”.^(^ ^21^ ^)^

On the other hand, even though homosexuality has not been considered a disease by the American Psychiatric Association (APA) since 1973 - making conversion therapy inappropriate and lacking in scientific support - 25% of physicians in our sample either believed conversion was possible or answered that they did not know, and almost 40% of them did not know that homosexuality is not considered a disease. Moreover, the American Medical Association condemns “reparative therapies” which seek to convert homosexuals to heterosexuals.^(^ ^25^ ^)^

The fact that a significant number of physicians are unaware of aspects that distinguish gender identity from sexual orientation stands out. One out of four physicians does not know if, or equivocally believes that, “most homosexuals want to be members of the opposite sex” (Item 3). This lack of knowledge was also clear on the item that reads “a homosexual person's gender identity does not agree with his/her biological sex” (Item 13) - only 44.6% of participants marked the correct answer. That shows that the majority of participants did not know that homosexuality represents sexual desire (affective and/or sexual attraction for people of the same sex), and not a gender dysphoria (strong desire to belong to the other gender group), which is the current accepted term according to the Diagnostic and Statistical Manual of Mental Disorders, 5th Edition (DSM-5).^(^ ^26^ ^)^ These two manifestations are different and require healthcare professionals to take different approaches due to their specificities.

We also found that about 33% of respondents believed that homosexuality is a personal choice. It is noteworthy that the belief that homosexuality is a personal choice is linked to higher levels of homophobia^(^ ^12^ ^)^

Different studies have found that scores of knowledge about homosexuality are generally lower among men and religious people.^(^ ^13^ ^,^ ^16^ ^)^ A study with physicians and students conducted in Serbia evaluated the knowledge about homosexuality and the attitude of participants regarding homosexuals. The study found that the individuals who were most knowledgeable about the subject were the ones with the least negative attitudes towards homosexuals.^(^ ^13^ ^)^ In the present study, no significant differences in knowledge were found between men and women, which may be a bias of the sample selection type (snowball).

Even though there are studies that suggest that a better knowledge of homosexuality can lead to more positive attitudes towards sexual minorities,^(^ ^13^ ^,^ ^14^ ^)^ the reality of some Brazilian universities seems to be very far from that. A study evaluating undergraduate medical students from public and private universities in the Brazilian city of Teresina, State of Piauí, showed sexuality classes essentially revolve around organic and pathological aspects and pay little attention to themes like sexual orientation, homophobia and gender roles.^(^ ^27^ ^)^

Another study evaluated 455 undergraduate medical students from the *Faculdade de Medicina de Botucatu,* in the State of São Paulo, and found that only 59% of male students knew that homosexuality is not a disease, while that number reached 80% among the female students. The item “a lesbian would prefer a man, if he were a real man and used the correct technique” was believed to be true, marked ‘I do not know’ or left blank by 38% of male and female students. In the evaluation where the students were divided according to their time in the course (first or second year, third or fourth year, and fifth or sixth year), no differences were found, among male or female students, regarding the knowledge that homosexuality is not a disease. That suggests that a medical student may graduate without that knowledge, despite the limitations mentioned by the authors of theirs being a cross-sectional and not a longitudinal study.^(^ ^28^ ^)^

One of the main limitations of our research is related to the snowball sample selection type, which could mean the sample is not representative of physicians in the Federal District. Studies with randomized samples may minimize possible selection biases. Moreover, longitudinal studies would be more recommended to evaluate the impact that better information about homosexuality would have on the attitude of medical staff and on the quality of clinical care provided to gays, lesbians and bisexuals. The application of the KHQ in this sample of physicians from the Federal District showed that several physicians are not familiar with several aspects of homosexuality. That may be related to more negative attitudes towards sexual minorities. Educational interventions during medical school and for graduated professionals may minimize this problem. Further studies are necessary to better understand the profile of physicians in relation to the LGBT population and study manners of educational interventions in order to minimize these problems.

## CONCLUSION

The adaptation and application of the Knowledge about Homosexuality Questionnaire in a sample of physicians from the Federal District of Brazil showed important aspects of their lack of knowledge about elementary characteristics of homosexuality and its related situations. The availability of a comprehensive and specific questionnaire about sexual orientation will allow more studies to be conducted with other samples (physicians and other healthcare professionals) and yield policies that widen the knowledge of the professionals, thus bringing improvements to the medical care of this population.
